# A novel 1p36.2 located gene, *APITD1*, with tumour-suppressive properties and a putative p53-binding domain, shows low expression in neuroblastoma tumours

**DOI:** 10.1038/sj.bjc.6602083

**Published:** 2004-08-24

**Authors:** C Krona, K Ejeskär, H Carén, F Abel, R-M Sjöberg, T Martinsson

**Affiliations:** 1 Department of Clinical Genetics, Institute for the Health of Women and Children, Göteborg University, Sahlgrenska University Hospital-East, SE-41685 Gothenburg, Sweden; 2 Cell and Gene Therapy Group, University of Melbourne, Murdoch Children's Research Institute, Royal Children's Hospital, Parkville VIC-3052, Australia

**Keywords:** 1p36, neuroblastoma, *TP53*, *APITD1*, TFIID

## Abstract

Neuroblastoma is characterised by a lack of *TP53* mutations and no other tumour suppressor gene consistently inactivated has yet been identified in this childhood cancer form. Characterisation of a new gene, denoted *APITD1*, in the neuroblastoma tumour suppressor candidate region in chromosome 1p36.22 reveals that *APITD1* contains a predicted TFIID-31 domain, representing the TATA box-binding protein-associated factor, TAF_II_31, which is required for p53-mediated transcription activation. Two different transcripts of this gene were shown to be ubiquitously expressed, one of them with an elevated expression in foetal tissues. Primary neuroblastoma tumours of all different stages showed either very weak or no measurable *APITD1* expression, contrary to the level of expression observed in neuroblastoma cell lines. A reduced pattern of expression was also observed in a set of various tumour types. *APITD1* was functionally tested by adding *APITD1* mRNA to neuroblastoma cells, leading to the cell growth to be reduced up to 90% compared to control cells, suggesting APITD1 to have a role in a cell death pathway. Furthermore, we determined the genomic organisation of *APITD1*. Automated genomic DNA sequencing of the coding region of the gene as well as the promoter sequence in 44 neuroblastoma tumours did not reveal any loss-of-function mutations, indicating that mutations in *APITD1* is not a common abnormality of neuroblastoma tumours. We suggest that low expression of this gene might interfere with the ability for apoptosis through the p53 pathway.

The chromosomal region 1p36 is commonly deleted in various paediatric soft tissue tumours including neuroblastoma (Brodeur *et al*, 1977, 1981), Wilms' tumour (Grundy *et al*, 1994; Steinberg *et al*, 2000), embryonal rhabdomyosarcoma (Bridge *et al*, 2000), pheochromocytoma (Benn *et al*, 2000) and germ cell tumours (Rodriguez *et al*, 1992; Stock *et al*, 1995; Bussey *et al*, 1999; Mostert *et al*, 2000; Schneider *et al*, 2001). Pheochromocytomas and neuroblastomas are both tumours originating in cells derived from the neural crest. We have previously narrowed down the shortest region of overlap of deletions (SRO) in our set of neuroblastoma tumours to 25 cM, between the markers D1S244 and D1S80 on chromosome 1, through loss of heterozygosity (LOH) studies of a large number of cases (Martinsson *et al*, 1995, 1997). A partly overlapping combined neuroblastoma and germ cell tumour SRO of 5 cM was later defined through the inclusion of a teratoma tumour with an interstitial 1p deletion (Ejeskär *et al*, 2001). Further evidence for the localisation of a neuroblastoma tumour suppressor gene to this region came from the finding of a 500 kb homozygous deletion within our SRO in a neuroblastoma cell line (Ohira *et al*, 2000). Mutation analysis of the coding sequences of all known genes within this 500 kb region indicated a low frequency of amino-acid changes, implying the presence of yet unidentified genes in the region which might be involved in the development and/or progression of neuroblastoma tumours (Ejeskär *et al*, 2000; Abel *et al*, 2002; Krona *et al*, 2003).

In this paper, we have, by means of *in silico* mapping, identified a gene coding for a domain with similarity to the human TATA box-binding protein-associated factor TAF_II_31 (locus name *TAF9*). The gene is localised to the 500 kb hot-spot region in 1p36.2, between *PGD* and *CORT*. TAF_II_31 has been identified as a critical protein required for p53-mediated transcriptional activation (Lu and Levine, 1995). Based on the localisation of this predicted gene, denoted *APITD1* (APoptosis-Inducing, TAF9-like Domain 1), and the fact that *TP53* mutations are rare in neuroblastoma tumours (Imamura *et al*, 1993; Komuro *et al*, 1993; Vogan *et al*, 1993; Hosoi *et al*, 1994), we hypothesised that loss of function for *APITD1* could be a way for tumour cells to overcome the cell growth-regulating properties of the p53 pathway.

In conclusion, we have characterised a new gene, which might be a member of the proteins involved in cell cycle regulation. Reduced levels of transcription from this gene, *APITD1*, present as an option for the cell to escape normal regulation during tumorigenesis.

## MATERIALS AND METHODS

### *In silico* mapping

In order to identify a putative gene in the neuroblastoma tumour suppressor gene candidate region on chromosome 1p36.22, UCSC Genome Browser August 2001 chromosome 1 draft sequence (URL:http://genome.ucsc.edu) was examined for transcripts, suggested by more than one gene prediction program, within the 500 kb tumour suppressor gene candidate region. Sequence alignment, open reading frame (ORF) prediction and primer selection were performed using the DNASTAR sequence analysis software (LASERGENE, Madison, WI, USA), or the OLIGO primer analysis software v.6 (Molecular Biology Insights, Cascade, CO, USA). For promoter analysis, genomic sequence from the UCSC contig assembly was analysed using the BDGP Neural Network Promoter Prediction program (URL:http://www.fruitfly.org/seq_to ols/promoter.html), and the CpG island prediction function of the MethPrimer program (URL:http://itsa.ucsf.edu/~urolab/m ethprimer) (Li and Dahiya, 2002). In order to analyse conservation of the *APITD1* gene, the TIGR gene indices (URL:http://www.tigr.org), which contain genomic sequences from a variety of organisms, were searched for expressed sequences translated in all reading frames, with significant similarity to the predicted protein from *APITD1*.

### RNA purification

Total RNA was extracted from neuroblastoma tumour samples using the Qiagen RNeasy kit for isolation of total RNA from animal tissues (QIAGEN, Valencia, CA, USA). For extraction of total RNA from cell pellets of neuroblastoma cell lines containing approximately 10^7^ cells, the Qiagen RNeasy kit for isolation of total RNA from animal cells was used. An additional DNase I digestion procedure (QIAGEN) was included in the isolation of RNA to remove contaminating DNA according to the instructions in the protocol.

### Reverse transcription

cDNA from neuroblastoma tumour samples and cell lines was made using SuperScript™ II Reverse Transcriptase (Invitrogen, Carlsbad, CA, USA), following the protocol for first-strand synthesis with 1 *μ*g of total RNA and 0.5 *μ*l of random hexamers (Promega, Madison, WI, USA).

### Northern blot

Total RNA (10 *μ*g) from the neuroblastoma cell lines SK-N-AS and SH-SY5Y was subjected to Northern blotting using the NorthernMax™ Kit (Ambion, Inc., Austin, TX, USA) and nylon membranes (Ambion). Primer sequences for amplification of an *APITD1* probe were chosen with the forward primer in exon 2 (5′-AAACAGACCATTGCGGCCATT-3′) and the reverse primer in exon 5 (5′-TTCCAAGCGGCGAGGG-3′); thus, the probe was complementary to both transcript versions of *APITD1*. Proper normalisation of the relative abundance of total RNA in the panel was assessed using primers for amplification of beta actin (*ACTB*): (forward primer) 5′-TCATGAAGTGTGACGTTGACATCCGT-3′ and (reverse primer) 5′-CCTAGAAGCATTTGCGGTGCACGATG-3′. The primer pairs were synthesised by Invitrogen. cDNA from SK-N-AS was amplified with these primer pairs with an initial denaturation at 95°C for 1 min, followed by amplification for 35 cycles of 30 s at 95°C, 30 s at 55°C and 2 min at 72°C. The PCR products were separated on an agarose gel and amplification products of the appropriate length were cut out from the gel and purified using the QIAquick gel extraction kit (QIAGEN). The hybridisation probes were then labelled with [alpha-^32^P]cytosine using the Megaprime™ DNA labelling system (Amersham, Piscataway, NJ, USA). Hybridisation was performed overnight at 42°C, and the film was exposed for 3–4 days before detection after hybridisation with *APITD1* probe and 1 day after hybridisation with the *ACTB* probe.

### Semiquantitative expression analysis

**Figure 1:**
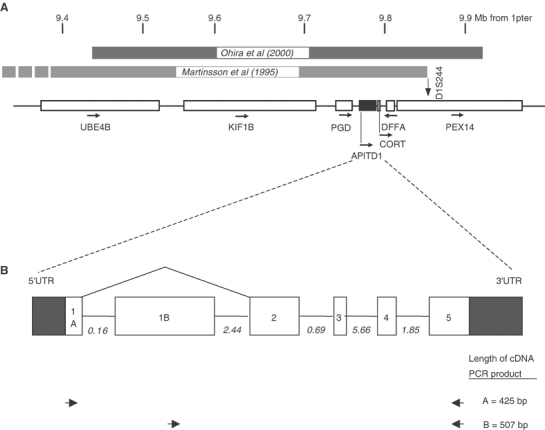
(**A**) Map of the 1p36.2 neuroblastoma tumour suppressor gene candidate region. The location of the genes is based on data from the UCSC genome browser; April 2002 draft sequence. The homozygously deleted region on chromosome 1p in a neuroblastoma cell line, found by Ohira *et al* (2000), is indicated by a dark shaded box, while the neuroblastoma SRO as defined by our group (Martinsson *et al*, 1995) is shown as a light grey box. The region includes the genes *UBE4B*, *KIF1B*, *PGD*, *APITD1*, *CORT*, *DFFA* and *PEX14*. *ICAT* is located just distal of the region. *APITD1* is marked with a black box. Arrows indicate the transcriptional direction of the genes. The scale in the top is the approximate distance from the 1p-terminal, in mega base pairs. (**B**) The genomic organisation of the *APITD1* gene, with the two alternative first exons, is shown with intron sizes indicated in kb. The location of the primers used for amplification of transcripts A and B from cDNA for RT–PCR expression analysis is also illustrated.

The tissue-specific expression of the two transcripts of the *APITD1* gene was analysed by amplification of commercially available cDNA from CLONTECH's Multiple Tissue cDNA Panels (CLONTECH, Palo Alto, CA, USA) and from OriGene (OriGene Technologies, Inc., Rockville, MD, USA), using two primer pairs. The primer pairs are shown in Figure 1

and listed in Table 1

**Table 1:** PCR primers used in this study

**Amplified sequence**	**Primer**	**Primer sequence**	**Length of fragment (bp)**	**Annealing T**
*APITD1-Promoter I*	FP	5′-CTTGTGGTCCCAGCTACTTG-3′	452	52
	RP	5′-TTCCAGTCGGGTGTAGAGG-3′		
*APITD1-Promoter II*	FP	5′-AGGTCACGGGTCGTTCACTC-3′	469	53
	RP	5′-GCGGCCAGGTCGGATT-3′		
*APITD1-Exon 1A*	FP	5′-CGTGTTTCGCGGAGAGTGGTGCTC-3′	566	65
	RP	5′-CCAGCGCAGGCCAGGGTCCAG-3′		
*APITD1-Exon 1B I*	FP	5′-GTGCTGGGAGGCGGTTTC-3′	438	65
	RP	5′-GCCCGCAGTTACCCTGACAA-3′		
*APITD1-Exon 1B II*	FP	5′-GGCCCAGAGCTCGTCCTTAGATGT-3′	514	55
	RP	5′-TAAAATGTAAAGGCGAAGTGTCAA-3′		
*APITD1-Exon 2*	FP	5′-ACTTGAAAAGCCACTCAGCCATTA-3′	400	55
	RP	5′-ACGAAGAACGCATGAGGATGATAA-3′		
*APITD1-Exon 3*	FP	5′-AAGGGTGAAGGGCTCTGGGAAGGA-3′	429	58
	RP	5′-CACCGTGATTGAAGGAGGCGTCAT-3′		
*APITD1-Exon 4*	FP	5′-TAATTGGGGTCTTAGGAACTTCAC-3′	434	55
	RP	5′-ATTGGCTTAGCAAGATCCATCATA-3′		
*APITD1-Exon 5 I*	FP	5′-TAACCCAGGCTTTGGCTTTTCTAC-3′	448	58
	RP	5′-AGCTTTGGCAGTTAACAATGTGGA-3′		
*APITD1-Exon 5 II*	FP	5′-CCGCTTGGAAAGTGCAGCCTTCTA-3′	462	58
	RP	5′-CATATGCCCTTTTACCTGGTGACC-3′		
*APITD1-Expr 1 (exon 1A-1B)*	FP	5′-GAGGAGCAGCAGCGATTCTC-3′	584	—*
	RP	5′-CGCGTTTCTTCAGTCCGTC-3′		
*APITD1-Expr 2 (exon 1A-1B)*	FP	5′-AGGGCGGAGCTGCGTACTTT-3′	208	—*
	RP	5′-GGTCCAGCCGCGTTGC-3′		
*APITD1-Expr 3 (exon 1A-3)*	FP	5′-AGGAGGAGGCGGAGA-3′	202	54
	RP	5′-GCAAACATTTCAAGGTCTTT-3′		
*APITD1-Expr 4 (exon 2-3)*	FP	5′-GGGTTGTCTTTGCGAGG-3′	128	54
	RP	5′-TTGCAAACATTTCAAGGTCT-3′		
*APITD1-Expr 5 (exon 2-4)*	FP	5′-GCTAAAGGCAGCAGTTCAC-3′	213	55
	RP	5′-CTCCTCCTGGCTAAGAGCTT-3′		
*APITD1-Expr 6 (exon 4-5)*	FP	5′-AGATGTGAAGCTCTTAGCCA-3′	190	54
	RP	5′-CGGCGAGGGACTTTA-3′		
*APITD1-Expr 7 (exon 4-CORT)*	FP	5′-ATTTTGCCAAAGACCTTGA-3′	531	—*
	RP	5′-TGTCATTACACTTGCGTGAG-3′		
*APITD1-Expr 8 (exon 5-CORT)*	FP	5′-TAAAGTCCCTCGCCGCTTGG-3′	133	—*
	RP	5′-AGGAGGCCGGGGGACAAT-3′		
*APITD1-Expr A (exon 1A-5)*	FP	5′-CGAGGAGCAGCAGCGATTCT-3′	425	54
	RP	5′-CGGCGAGGGACTTTA-3′		
*APITD1-Expr B (exon 1B-5)*	FP	5′-ACTGAAGAAACGCGGGAATG-3′	507	54
	RP	5′-CGGCGAGGGACTTTA-3′		

Column 1: amplified sequence; *Promoter I* and *Promoter II* are overlapping primer pairs, which together cover 800 bp before the initiation codon in transcript A; *Exon 1B I* and *Exon 1B II* cover the starting exon (1B) in transcript B together; *Exon 5 I* and *Exon 5 II* cover exon 5 and approximately 500 bp into the 3′ UTR region; *APITD1-Expr* primers are located in the exons indicated within the parantheses, and they were used for amplification of cDNA. Column 5: Annealing temperature for PCR amplification

* No PCR product was obtained with the *APITD1-Expr 1*, *-Expr 2*, *-Expr 7* and *-Expr 8* primers at various temperatures, implying that exons 1A and 1B are not in the same transcript, and that *APITD1* is not in the same transcription unit as the nearby located gene *CORT*.

as *APITD1-Expr* A-B. Amplification of *ACTB* was performed as an internal control with the same primer pair that was used for amplification of the *ACTB* probe for Northern blotting. PCR assays were performed with an initial denaturation at 95°C for 1 min, followed by amplification for 30 cycles (*ACTB*) or 35 cycles (*APITD1* transcripts) of 30 s at 95°C, 30 s at 54°C and 2 min at 72°C. All reactions were conducted in a total volume of 50 *μ*l. The expression of *APITD1* was also determined in cDNA reverse transcribed from various primary tumour samples and neuroblastoma cell lines (SK-N-AS, IMR-32, SK-N-F1, SK-N-BE(2) and SH-SY5Y). Additional reactions were performed with control tissues and primer pairs located in varying parts of the predicted transcripts, in order to determine which exons are present in the mature mRNA (Table 1; *APITD1-Expr 1–6*). We have also analysed the possibility of *APITD1* being a part of the adjacent gene *CORT*. Therefore, two primer pairs were designed, with the forward primer located in *APITD1* exon 4 or 5, respectively, and with the reverse primers in the coding sequence of *CORT* (Table 1; *APITD1-Expr 7–8*). These primer pairs were used in attempts to amplify cDNA by reverse transcriptase–PCR (RT–PCR) at different PCR conditions.

### Real-time PCR expression analysis

Primers and probes for amplification of both *APITD1* transcripts were designed spanning intron 1. They were ordered through the Applied Biosystems ‘TaqMan® Assays-by-Design^SM^ Gene Expression Service’ facility (URL:http://myscience.appliedbiosys tems.com). TaqMan primers and probe for *GUSB* (*β*-glucuronidase) were derived from Applied Biosystems ‘TaqMan® Assays-on-Demand™ Gene Expression Products’ and amplification of *GUSB* was used as an internal control for the integrity and relative amount of mRNA. The real-time PCR reactions were performed using an ABI PRISM® 7900HT Sequence Detection System (Applied Biosystems, Foster City, CA, USA). The mean *C*_T_ values for duplicate samples of neuroblastoma cell lines or neuroblastoma tumours were used to calculate the amount of both transcripts from a standard curve. The calculated expression levels were then normalised to the amount of *GUSB* in each sample in order to achieve a relative expression amount. This relative expression amount of both transcripts in a group of four stage 2 tumours from children with no residual evidence of disease was compared with a two-sample *t*-test to a group of six stage 3 or stage 4 tumours from children who had died of the disease.

### Hybrid panel mapping

In order to assure the chromosomal localisation of the *APITD1* gene, we used the NIGMS human/rodent somatic cell hybrid mapping panel #2, version 3 (Coriell Institute, Camden, NJ, USA). The primer pair for amplification of exon 4 (Table 1; *APITD1-Exon 4*) was used in amplification reactions, with 100 ng DNA from hybrid cells representing all human chromosomes, for 30 cycles (30 s at 95°C, 30 s at 55°C and 1 min at 72°C). PCR products were then loaded onto a 2% agarose gel, stained with ethidium bromide and visualised under UV illumination.

### *In vitro* transcription of mRNA

DNA fragments of the coding region of transcript A from *APITD1* linked to the Sp6 promoter sequence and a polyA tail at the end of the fragment were constructed by PCR using primers: (forward primer) 5′-ATTTAGGTGACACTATAGAAGATTGCCCAGGGTCGGCCCG CAGTGATG-3′ and (reverse primer) 5′-GG(T)_30_ ACAATGTGGAGAATTCAGCAC-3′, with IMAGE clone 4051739 (gene bank locus: BC029430) as a template. The PCR was performed using High Fidelity Taq (Roche Diagnostics, Mannheim, Germany) according to the supplier's protocol, with an annealing temperature of 60°C and 30 cycles of PCR. In all, 500 ng of the PCR product (663 bp) was used as a template for *in vitro* mRNA using mMESSAGE mMachine (Ambion). The procedure was according to the supplier's protocol using 4 h incubation at 37°C. The mRNA was DNase I treated for 15 min at 37°C and LiCl-precipitated before size and concentration evaluation by agarose gel electrophoresis. The control green fluorescent protein (*GFP*) mRNA was produced using 500 ng of *Eco*RI-digested Sp6-EGFP vector as template in the *in vitro* mRNA reaction. The concentrations of *GFP* mRNA and *APITD1*A mRNA were adjusted to be equal and evaluated on agarose before each further experiment.

### Cell culture

The neuroblastoma cell lines SK-N-BE(2) and SK-N-AS (American Type Culture Collection (ATCC), Manassas, VA, USA), the embryonic kidney derived cell line 293 (ATCC) and the lymphoblast cell line K562 (ATCC) were cultured in Dulbecco's modified Eagle medium (Invitrogen) with 10% foetal calf serum (Sigma-Aldrich Corp., St Louis, MO, USA) in 37°C using standard procedures. Cells with 60–80% confluence were used in transfection experiments. Immediately before the transfection procedure, the SK-N-BE(2) cells, the SK-N-AS cells and the 293 cells were trypsinised and washed twice with PBS, and the cell numbers and the viability were calculated using trypan blue staining. The suspension cells K562 were used only if the viability was 90% or more at the day of transfection.

### Transfection

The transfection experiments were performed in a 12-well plate using 4 × 10^5^ cells well^−1^ for SK-N-BE(2), SK-N-AS, and K562 cells and 7 × 10^5^ cells well^−1^ for 293 cells, all in 2 ml Dulbecco's modified Eagle medium with 10% foetal calf serum per well. For each transfection, a total of 1.5 *μ*g of mRNA (A: 1.5 *μ*g *GFP* mRNA; B: 0.3 *μ*g *APITD1*A mRNA+1.2 *μ*g *GFP* mRNA; C: 1.5 *μ*g *APITD1*A mRNA) and 5 *μ*l Lipofectamine-2000 (Invitrogen) were used. The mRNA and the Lipofectamine-2000 were incubated for 30 min in 200 *μ*l serum-free medium (Opti-MEM, Invitrogen) before being added to the cell suspensions. The cell and Lipofectamine-2000–mRNA complex mix was immediately divided into 96-well plates at 10^4^ cells well^−1^ (SK-N-BE(2), SK-N-AS, K562) or 1.7 × 10^4^ cells well^−1^ (293) (50 *μ*l well^−1^), and incubated at 37°C for 5 h before an extra 50 *μ*l well^−1^ of Dulbecco's modified (Eagle) medium with 10% foetal calf serum was added. The cells were incubated for 1–4 days at 37°C for cell growth studies.

### Cell growth studies

After incubation for 1, 2, 3 and 4 days, respectively, at 37°C, 20 *μ*l of CellTiter96 AQ_ueous_ One Solution Cell Proliferation Assay (Promega) was added to each 96-well plate with 100 *μ*l cell suspension. For each experiment, four separate wells were tested at each time point. The mix was incubated in the dark at 37°C for 4 h before the absorbance at 490 nm was evaluated in an ELISA reader. The cell number in each well was calculated using a standard curve with known cell numbers using the same procedures as above. The mean number of cells per well was calculated for each experiment at each time point. At 1 day after transfection, the transfection efficiency was evaluated using fluorescence microscopy, by counting the number of green cells in the *GFP* mRNA experiments. Experiments comparing different mRNA:s were performed from the same batch of cells and treated equally all through the experimental procedure.

### DNA sequencing

Genomic DNA extracted by standard procedures from 44 frozen (−70°C) primary neuroblastoma tumours of all different stages was used for sequencing analysis. The tumours were staged according to the International Neuroblastoma Staging System criteria (Brodeur *et al*, 1988, 1993). Four were stage 4S, four were stage 1, three were stage 2a, one was stage 2b, 13 were stage 3, 15 were stage 4 and four were of unknown stages. Of the stage 3 and 4 tumours, 16 had 1p deletion, as determined by FISH and/or microsatellite analysis. DNA was also extracted from EDTA blood, obtained from control individuals. The sequence results from the tumour samples were compared to the reference sequences from the UCSC Genome Browser (chr1_29_927.b and chr1_29_927.g). Control samples were also included in the sequencing of all exons, according to Table 2

**Table 2:** DNA variations in *APITD1* sequence

		**Tumours**				**Controls**			
**DNA variation**	**Genomic location**	**Screened number**	**HetZ**	**HomZ/HemZ for non-ref. seq. allele**	**HomZ/HemZ for ref. seq. allele**	**Screened number**	**HetZ**	**HomZ for non-ref. seq. allele**	**HomZ for ref. seq. allele**
c.1-464 T>C	Promoter^a^	44	30 (68%)	6 (14%)	8 (18%)	38	18 (47%)	8 (21%)	12 (32%)
c.1-290 T>C	Promoter^a^	44	29 (66%)	6 (14%)	9 (20%)	42	14 (33%)	14 (33%)	14 (33%)
c.1-225 G>A	Promoter^a^	44	1 (2%)	0	43 (98%)	42	0	0	42 (100%)
c.1-53 C>T	Promoter^a^	44	2 (5%)	0	42 (95%)	38	1 (3%)	0	37 (97%)
c.1-36 G>C^b^	5′ UTR^c^	44	10 (23%)	0	34 (77%)	38	7 (18%)	2 (5%)	29 (76%)
c.1-25 G>C^b^	5′ UTR^c^	44	10 (23%)	0	34 (77%)	38	7 (18%)	2 (5%)	29 (76%)
c.1-644 A>G	5′ UTR^d^	44	29 (66%)	8 (18%)	7 (16%)	48	12 (25%)	21 (44%)	15 (31%)
c.1-316 A>G	5′ UTR^d^	44	10 (23%)	34 (77%)	0	46	2 (4%)	42 (91%)	2 (4%)
c.277-(14-5) insT	Intron 4^b^	44	6 (14%)	0	38 (86%)	43	10 (23%)	0	33 (77%)
c.665 A>G	3′ UTR^c^	44	10 (23%)	33 (75%)	1 (2%)	46	7 (15%)	37 (80%)	2 (4%)

a The UCSC Genome Browser August 2001 Chromosome 1 draft sequence was used as a reference sequence, with the A in the initiation codon starting at base 135 in transcript A (chr1_29_927.b) denoted nucleotide 1.

b Linkage disequilibrium is present between the two variations.

c Transcript A was used as a reference.

d Transcript B (chr1_29_927.g) was used as a reference. Base number 1 was assigned to the A base of an initiation codon starting at base 736 in the transcript. Column 3: number of tumour samples screened for each variation. Columns 4–6: number of tumour samples with each observed allele and frequency of the allele in the screened population; HetZ, heterozygote; HomZ, homozygote; HemZ, hemizygote; ref., reference; seq., sequence. Column 7: number of control samples screened for each variation. Columns 8–10: number of control samples with each observed allele and frequency of the allele in the screened population; HetZ, heterozygote; HomZ, homozygote; ref., reference; seq., sequence.

. Primers for amplification of the coding sequences of the gene as well as the predicted promoter sequences are listed in Table 1. The concentration of PCR products was estimated by comparison to a low DNA mass ladder (Invitrogen) on an agarose gel. The PCR products were purified with ExoSAP-IT (USB, Corp., Cleveland, OH, USA). The sequencing reactions were made using ABI PRISM Big Dye Terminator Cycle Sequencing Ready Reaction Kit (Applied Biosystems) according to protocols. Sequencing was performed using an ABI PRISM 3100 DNA Sequencer (Applied Biosystems). Sequence analysis was conducted using the Vector NTI sequence analysis software (InforMax, Frederick, MD, USA). In cases where a deviation from the reference sequence was observed, it was confirmed through re-sequencing from the opposite direction.

## RESULTS

### Genomic organisation of *APITD1*

**Figure 2:**
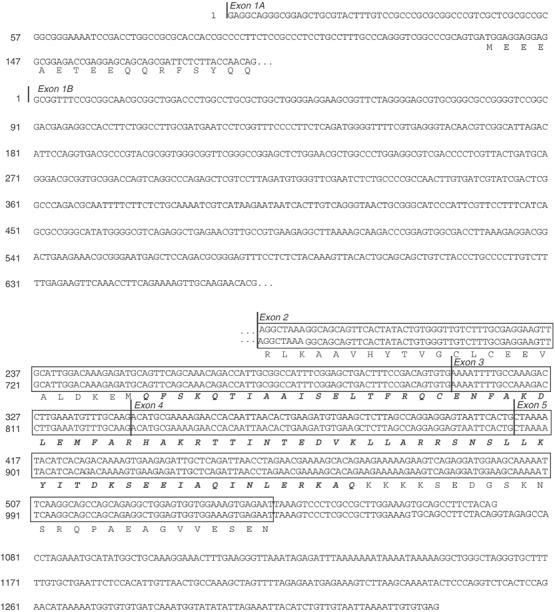
The two alternative transcript versions of the *APITD1* gene. Both transcripts share exons 2–5, but they differ in the starting exons and in the 3′UTR sequences. Exon 1A is located approximately 160 bp upstream of exon 1B in the genomic sequence. The translated sequence from the ORF in transcript A and the in-frame ORF in transcript B, which starts immediately prior to the putative TFIID-31 domain, is shown. The amino-acid sequence with significant similarity to the TFIID-31 domain is marked in bold face and italics. The last 622 bp of transcript A (chr1_29_927.b), which consists of exon 2 from the nearby located gene *CORT* and noncoding genomic sequence, is not shown.

**Figure 3:**
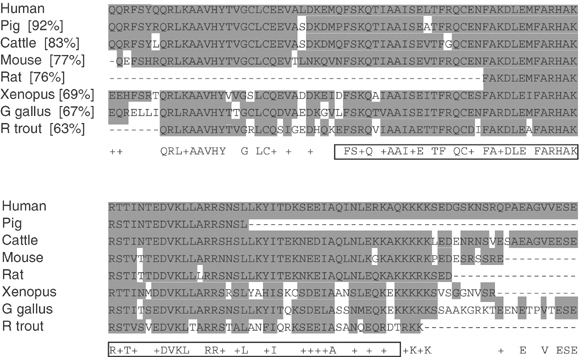
Conserved amino acids in the translated *APITD1* transcript A ORF. The first nine amino acids in the translated ORF did not align to the translated expressed sequences from other organisms in the TIGR database, and they are not shown. Percent identity with the human sequence is shown within brackets after the name of each organism. Residues which are identical or chemically similar to the human amino-acid sequence are highlighted in grey. The residues which are identical or similar among all aligned sequences are indicated under the alignment, and the TFIID-31 similar domain is framed.

A gene was predicted to reside distal to the *CORT* gene but proximal to the *PGD* gene, in the neuroblastoma tumour suppressor gene candidate region on chromosome 1p36.22. Due to similarity to the TFIID-31 domain in the transcription initiation factor TAF_II_31 (locus name *TAF9*), it was denoted *APITD1* (APoptosis-Inducing, TAF9-like Domain 1). Two alternative versions of the gene were proposed from the predicted mRNA sequences at the UCSC Genome Browser. A closer examination of a 1214 bp long predicted mRNA, chr1_29_927.b (*APITD1* transcript A) showed that the sequence between bases 588 and 1111 is identical to exon 2 of the nearby located gene *CORT* and the last 103 bases in the predicted transcript between bases 1112 and 1214 is probably not a transcribed genomic sequence. Using RT–PCR, we demonstrated that exon 2 of *CORT* is not included in the *APITD1* transcript A; thus a 587 bp long transcript remains (Figure 2

). A 1331 bp long transcript, chr1_29_927.g, with an alternative first exon was also predicted (*APITD1* transcript B) (Figure 2). Sequence alignment of these transcripts against genomic sequences from the public databases determined the genomic structure of *APITD1* to consist of six exons spread over approximately 13 kb (Figure 1). Two CpG islands with a GC content of more than 80% were identified preceding and overlapping the first exons of both transcript versions of the gene. A possible transcription promoter was predicted to reside approximately 200 bp prior to exon 1A. The longest ORF in transcript A stretches from base 135 to 548 and it encodes a hypothetical protein of 138 amino acids with a molecular weight of 16 kDa (Figure 2). The translation start site in this transcript is set within the correct context for translation initiation with a purine at −3 and G at +4 (Kozak, 1996). In transcript B, however, there is no ORF with the optimal translation initiation context. Two ORFs of adequate length exist in the transcript, both with a C at position +4 instead of the consensus nucleotide G. The first ORF is set between base 266 (in exon 1B) and 631. No related human sequences are retrieved through BLAST searches of nucleotide and protein databases, with the hypothetical protein translated from this ORF. The other ORF runs in the same reading frame as the ORF in transcript A; it is located between base 736 (in exon 2) and 1032 and it encodes for a hypothetical protein of 99 amino acids with a molecular weight of 11 kDa (Figure 2). This ORF, like the ORF in transcript A, encodes an amino-acid sequence with homology to the TFIID-31 subunit of the transcription initiation factor TFIID, required for p53-mediated transcription activation. The translated ORF in transcript A also displays a high sequence similarity to translated expressed sequences from other organisms than human (Figure 3

).

### Northern blot

**Figure 4:**
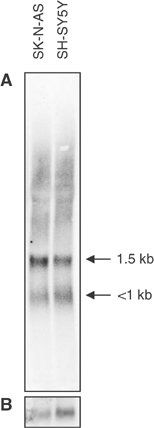
Transcript size of the *APITD1* gene. Northern blot analysis of total RNA isolated from the neuroblastoma cell lines SK-N-AS and SH-SY5Y. The blot was hybridised with a radio labelled probe. (**A**) *APITD1*. (**B**) *ACTB* internal loading control.

Northern blot hybridisation of neuroblastoma cell lines SK-N-AS and SH-SY5Y with use of a radio labelled *APITD1* probe identified two transcripts of approximately 1.5 and less than 1 kb respectively. The higher molecular weight transcript was more abundant (Figure 4

).

### Transcript variants from the *APITD1* gene

In order to establish which exonic sequences are transcribed from the *APITD1* gene, RT–PCR was performed on cDNA extracted from kidney, foetal kidney, brain, foetal brain and stomach, using primer pairs located in various exons (Table 1). Attempts to amplify the sequence between exons 1A and 1B failed, implying that these exons are located in different transcripts. All other combinations of amplification that are listed in Table 1 resulted in PCR products of the expected sizes (data not shown). There was no amplification product in the RT–PCR reactions when forward primers, located in *APITD1*, were used with reverse primers located in *CORT*. Thus, our data show that *APITD1* is a gene distinct from *CORT*.

### Tissue-specific expression

**Figure 5:**
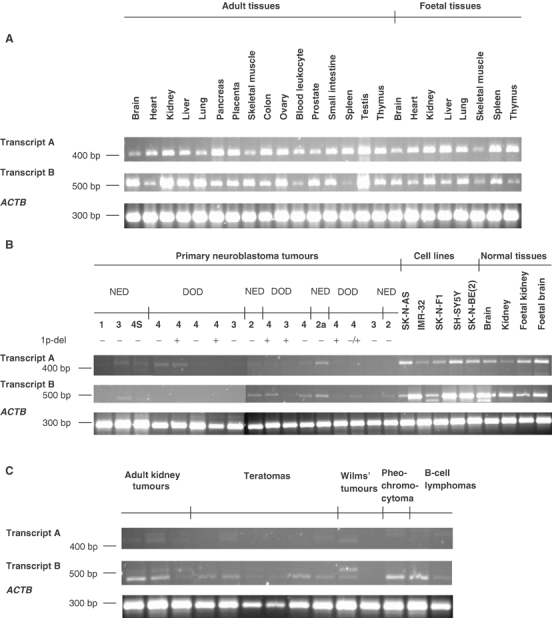
RT–PCR expression analysis of transcripts A and B in *APITD1*. Amplification of *APITD1* transcript A and *APITD1* transcript B in each sample is compared to the amplification of *ACTB*. (**A**) Fairly ubiquitous expression of both transcripts in a set of normal adult and foetal tissues (CLONTECH). Lanes 1–24, PCR products from the indicated tissues. (**B**) Reduced expression of *APITD1* gene products in neuroblastoma tumours of different stages compared to neuroblastoma cell lines and adult and foetal normal tissues (OriGene). The outcome of the patients, the stage of neuroblastoma and the status of chromosome 1p is indicated above the upper panel; NED, no evidence of disease; DOD, dead of disease; 1, 2, 2a, 3, 4 and 4S, stages of neuroblastoma; −, negative for 1p deletion; +, positive for 1p deletion; ±, uncertain result (based on FISH and microsatellite analysis). Lanes 1–17, neuroblastoma tumours; lanes 18–26, neuroblastoma cell lines and normal tissues as indicted above the panel. (**C**) Reduced expression of *APITD1* gene products in various tumours. Lanes 1–14, tumours of various types as indicated above the panel.

Both *APITD1* transcripts were ubiquitously expressed in all tested normal tissues, with an especially high level of expression in the adult testis of both transcripts and in the adult kidney of transcript B. A low level of expression of transcript B was detected in the peripheral blood leukocyte and spleen from adult tissues, and in the skeletal muscle and thymus from foetal tissues (Figure 5A

). The expression of transcript A was generally higher in foetal tissues than in adult tissues, while the opposite situation occurred for transcript B.

### *APITD1* expression in tumour samples and neuroblastoma cell lines

The *APITD1* transcripts were either not expressed or only present at low levels in both neuroblastoma tumours (Figure 5B) and a set of various tumour types (Figure 5C). A PCR product of length shorter than 507 bp was also observed in the amplification reaction of transcript B in some tumours. This product was analysed by sequencing, and was shown to be an amplification product originating from a gene on chromosome 16, with a partial sequence homology to the primer sequences (data not shown). The expression of both transcripts was markedly reduced compared to the level of expression in normal tissues and in the neuroblastoma cell lines, with the exception of IMR-32, which had a weak expression of both transcripts.

A more sensitive screening by quantitative real-time PCR revealed that the mean relative expression level of transcript A was significantly lower in the high-stage tumours (mean 0.07; s.d. 0.03) than in the low-stage tumours (mean 0.35; s.d. 0.17) (*P*=0.0022). There was also a significant difference (*P*=0.0016) in the relative mean expression levels of transcript B between the high-stage tumours (mean 0.74; s.d. 0.26) and the low-stage tumours (mean 1.85; s.d. 0.43). The relative amount of the *APITD1* transcripts was considerably higher in the cell lines than in the tumour samples (Table 3

**Table 3:** Amounts of *APITD1* transcripts measured by quantitative real-time PCR

**Cell lines**	***APITD1* A**	***APITD1* B**							
SK-N-AS	0.70	1.61							
SK-N-DZ	1.17	3.14							
SK-N-SH	2.07	3.73							
SK-N-BE(2)	2.06	2.41							
SK-N-F1	0.79	4.28							
SH-SY5Y	1.65	2.21							
									
**Cases**			**Group**	**Stage**	**1p-del**	**MYCN**	**17q gain**	**Ploidi**	**Outcome**
18F8	0.60	2.37	F	2A	neg			4n	NED
20S9	0.23	1.32	F	2	neg	neg			NED
23S4	0.36	1.77	F	2	neg	neg		3n	NED
25S9	0.22	1.92	F	2	neg	neg			NED
									
4F1	0.12	0.76	UF	4	neg	neg	pos	2n	DOD
10S2	0.08	0.45	UF	4	pos/neg	pos			DOD
13S0	0.06	1.00	UF	4	pos	pos	pos		DOD
13S1	0.09	0.39	UF	3	pos	pos	pos		DOD
15S3	0.06	0.89	UF	4	neg/pos	neg	pos		DOD
17S2	0.05	0.94	UF	4	neg	neg			DOD

Column 2: relative amount of *APITD1* A expression. Column 3: relative amount of *APITD1* B expression. Column 4: F, favourable; UF, unfavourable; based on prognostic markers and the outcome of the disease. Columns 6–8: 1p-del, 1p-deletion; MYCN, *MYCN* amplification; 17q gain, neg, negative; pos, positive; neg/pos; ambiguous results based on microsatellite marker analysis (according to Martinsson *et al*, 1995) and FISH, empty cells, not determined. Column 10: NED, no evidence of disease DOD, dead of disease.

).

### Hybrid panel mapping

**Figure 6:**
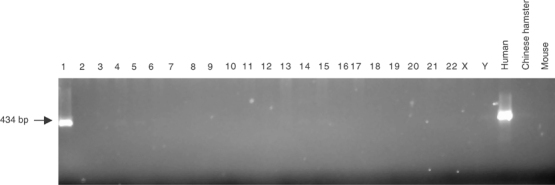
PCR amplification of *APITD1* exon 4 in a human/rodent somatic cell hybrid mapping panel. Amplification products of the appropriate size are obtained in the chromosome 1 cell hybrid and in the human cell line control exclusively.

PCR products of the expected size (434 bp) were obtained in the chromosome 1 cell hybrid and in the human cell line control exclusively (Figure 6

).

### Functional studies on cell growth

**Figure 7:**
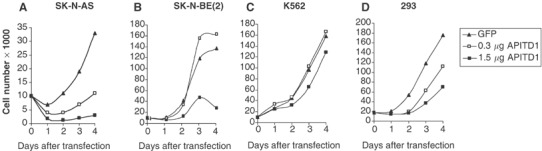
Comparative growth curves of cell lines after transfection of 1.5 *μ*g *GFP* mRNA; 0.3 *μ*g *APITD1*A mRNA+1.2 *μ*g *GFP* mRNA or 1.5 *μ*g *APITD1*A mRNA, respectively. (**A**) SK-N-AS (neuroblastoma). (**B**) SK-N-BE(2) (neuroblastoma). (**C**) K562 (lymphoblast). (**D**) 293 (transformed embryonal kidney).

**Figure 8:**
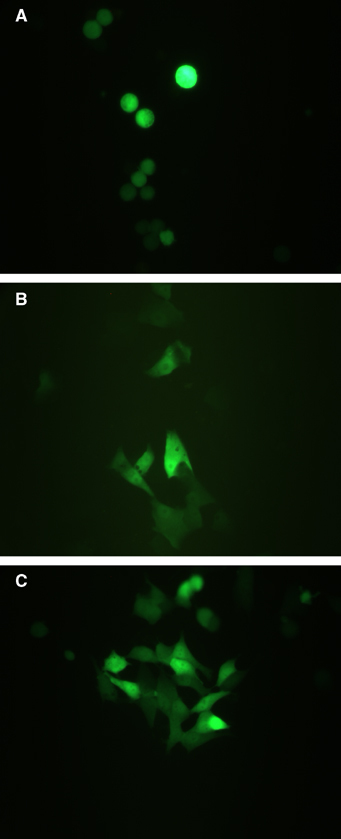
Expression of GFP in transfected cells shows that the mRNA has been introduced into the cells. (**A**) K562 (lymphoblast). (**B**) SK-N-AS (neuroblastoma). (**C**) SK-N-BE (neuroblastoma).

The mRNA transfection experiments showed that by adding 1.5 *μ*g of *APITD1*A mRNA to the neuroblastoma cell line SK-N-AS the cell number was reduced by 70% after 24 h, and by 90% after 2–4 days of incubation, compared to *GFP* mRNA-transfected cells. If 0.3 *μ*g *APITD1*A mRNA was mixed with 1.2 *μ*g of *GFP* mRNA, the reductions in cell numbers after 24 h were 40% and 65% after 2–4 days (Figure 7A

). After 2–3 days, the number of SK-N-BE(2) cells in the 1.5 *μ*g *APITD1*A-transfected experiment was 60–70% below the *GFP* control and after 4 days the reduction was 80%. The SK-N-BE(2) cells transfected with a mix of 0.3 *μ*g *APITD1*A mRNA and 1.2 *μ*g *GFP* mRNA showed an increase in cell numbers by 20–30% compared to the *GFP*-transfected cells after 3–4 days (Figure 7B). In K562 cells transfected with 1.5 *μ*g *APITD1*A mRNA, a slighter reduction in cell numbers of 20–30% could be seen after 2–4 days. Also, in this experiment, there was a slight increase in the number of cells transfected with the lower concentration of *APITD1*A mRNA compared to the *GFP*-transfected control cells (Figure 7C). In 293 cells, an equal reduction in cell numbers could be seen after 2 days (60–70%), for both *APITD1*A mRNA experiments compared to control cells. After 3–4 days of incubation, the experiment using 1.5 *μ*g *APITD1*A mRNA still showed 60–70% reduction, while the 0.3 *μ*g *APITD1*A mRNA experiment showed 40% reduction compared to control cells (Figure 7D). The transfection efficiency of *GFP* mRNA for SK-N-AS, SK-N-BE(2) and 293 cells was determined to 90% and for K562 to 60% fluorescence microscopy 24 h after transfection (Figure 8

).

### Mutation analysis of *APITD1*

No changes were found in the coding sequence of *APITD1* in the tumour samples when compared to the reference samples. Variations that were identified in noncoding sequence are listed in Table 2.

## DISCUSSION

Mutations in the tumour suppressor gene *TP53,* which has implications for tumorigenesis in many other tumour types (Nigro *et al*, 1989; Levine *et al*, 1991), are rare in primary neuroblastoma tumours (Imamura *et al*, 1993; Komuro *et al*, 1993; Vogan *et al*, 1993; Hosoi *et al*, 1994). A frequent characteristic of mainly advanced neuroblastoma tumours is deletion of the distal part of chromosome region 1p, indicating that this is the location of a neuroblastoma tumour suppressor gene (NBS; OMIM 256700). Our group and others have tried to narrow down the consensus region of the NBS locus (Caron *et al*, 1995; Maris *et al*, 1995; Martinsson *et al*, 1995, 1997; White *et al*, 1995; Ichimiya *et al*, 1999; Bauer *et al*, 2001; Caron *et al*, 2001; Ejeskär *et al*, 2001; Maris *et al*, 2001; Spieker *et al*, 2001; White *et al*, 2001). In 2000, Ohira *et al* presented the first case of a homozygous deletion in the region in a neuroblastoma cell line. The 500 kb deletion partly overlaps the most proximal part of the consensus LOH region determined by us in our set of tumours. We have previously screened all annotated genes within this 500 kb region for mutations in neuroblastoma patients (Ejeskär *et al*, 2000; Abel *et al*, 2002; Krona *et al*, 2003). In this study, we have further explored this genomic region by the characterisation of a predicted gene, *APITD1*.

The coding sequence of the predicted gene in the neuroblastoma tumour suppressor candidate region on chromosome 1p36.22 is transcribed into alternative 5′ end mRNAs. The predicted amino-acid sequence of this gene, which we denoted *APITD1* (Apoptosis-Inducing, TAF9-like Domain 1), is similar to the human TATA box-binding protein-associated factor TAF_II_31, a component of TFIID. TAF_II_31 has been identified as a critical protein required for p53-mediated transcriptional activation (Lu and Levine, 1995). The ATG start codon of the *APITD1* A transcript occurs in a strong context, RNNatgG, where R is a purine (Kozak, 1996), suggesting that translation from this mRNA is active. However, despite the fact that the B transcript was shown to be ubiquitously expressed, the translational status of this mRNA must be considered with caution since there is no translation initiation site headed by a strong ATG codon (Kozak, 1996) in this transcript.

We have shown that expression of *APITD1* transcript A is generally elevated in a set of eight foetal tissues compared with the same tissues from adults, with the most notable difference observed in cDNA extracted from brain and heart tissue, where the foetal expression was markedly elevated compared to the adult counterparts. Observed variations in band intensity should be reliably attributed to true differences in target mRNA abundance since the cDNA panel used has been previously normalised to the expression levels of at least four different housekeeping genes. Furthermore, replicate experiments resulted in comparable band intensities for each sample. The expression in neuroblastoma tumours of both transcripts was low when compared with neuroblastoma cell lines and normal tissues. Furthermore, a more sensitive quantification by real-time PCR revealed that the level of expression of both *APITD1* transcripts was significantly reduced in a group of high-stage tumours from patients who had died from the disease when compared to a group of low-stage tumours from patients with no residual evidence of disease. The relatively strong expression of *APITD1* transcript A in foetal tissues appears in strong contrast to the low expression in embryonal neuroblastoma tumours. We therefore propose that timed expression of *APITD1* is important for the proper differentiation of foetal cells. On the other hand, expression of both *APITD1* transcripts was also almost absent in a panel of other tumours than neuroblastoma, both adult (kidney) and paediatric (teratomas, Wilms' tumours, B-cell lymphomas and a pheochromocytoma). This indicates that *APITD1* can have a more general significance for regulation of the cell cycle in normal cells.

We could show that *APITD1*A mRNA has a strong effect on cell growth in neuroblastoma cells (SK-N-BE(2) and SK-N-AS). The effect was dose dependent in SK-N-AS cells where the number of viable cells was decreased by 90% 2 days after transfection in the *APITD1*A mRNA experiment compared to cells where an equal amount of *GFP* mRNA had been added. If four out of five of the *APITD1*A mRNA was exchanged to control mRNA, the decrease was 65% 2 days after transfection (Figure 7A). There was also a clear reduction in the number of viable cells when SK-N-BE(2) was transfected with 1.5 *μ*g *APITD1*A mRNA. However, when 0.3 *μ*g of *APITD1*A mRNA was used, the SK-N-BE(2) cells were even growing faster after 3–4 days than the control cells did (Figure 7B). The effect of *APITD1*A mRNA on the cells could be due to a number of different reasons, that is, increased apoptosis, growth arrest, or some general toxicity of the *APITD1*A mRNA. The amount of mRNA added to each cell can always be argued, but still these experiments indicate that *APITD1*A indeed can have a role in an apoptotic or growth suppression pathway in neuroblastoma cells.

An effect could also be seen on 293 cells by adding *APITD1*A mRNA, even though not quite as strong (60–70% reduction) as neuroblastoma cells (80–90% reduction) after 4 days incubation. This difference in effect can of course depend on a number of reasons, like the amount of *APITD1*A mRNA per cell and the specific properties of the cells of these particular cell lines. However, one interesting thing to note is that the dose of *APITD1*A mRNA does not seem to make a difference the first couple of days after transfection in 293 cells. The difference in effect from the different doses comes first 3 days after transfection (Figure 7D). A difference this long after transfection might be due to the ability of the cell to break down mRNA. In the experiment with five times as much *APITD1*A mRNA, there will probably be more mRNA molecules left longer in the cell; thus the *APITD1*A mRNA will have time to affect more cells. The lymphoblast cell line K562 shows minor differences in cell growth between *APITD1*A mRNA experiments and control mRNA experiments (Figure 7C). Thus, *APITD1*A mRNA does not seem to activate any cell death or growth suppression pathway in these cells, as it seems to do in the neuroblastoma cell lines and in the transformed embryonic kidney cell line 293. However, these are indeed very different types of cells, and they are likely to be defective in different cellular pathways.

Thus, we have shown that *APITD1*A mRNA is capable of inducing cell death or growth suppression pathways in two neuroblastoma cell lines. As indicated in our expression studies, the level of *APITD1* transcription in the neuroblastoma cell lines is intrinsically not as low as in the neuroblastoma tumours, which suggests that this is not the mechanism responsible for the immortality of neuroblastoma cell lines. Still, since the neuroblastoma cell lines are known to be different from primary tumours in many aspects (i.e. karyotype and milieu), it would not be surprising if they turned out to have different approaches than primary tumours to avoid the cell growth-regulating pathways of a normal cell.

Since APITD1 has a domain with homology to the human TATA box-binding protein-associated factor TAF_II_31, which has been identified as a critical protein required for p53-mediated transcriptional activation, expression of *APITD1* might be necessary for the induction of apoptosis in defective foetal cells by the p53 pathway. There is also high sequence similarity between the translated ORF of *APITD1*A and translated expressed sequences from other organisms. For instance, 77 out of 83 (92%) of the amino acids in a part of this hypothetical protein are identical to a translated nucleotide sequence from pig. By comparison, the alignment between the hypothetical TFIID-31 domain in APITD1 and the TFIID-31 domain in TAF_II_31 was only 57%. Thus, it seems that *APITD1* encodes an evolutionary conserved protein motive, similar to but different from TAF_II_31, in accordance with our hypothesis that APITD1 is a new member of the family of important proteins involved in cell regulation. In conclusion, we have characterised a novel gene, encoding a domain similar to a p53 transcription initiation factor, in the neuroblastoma tumour suppressor gene candidate region on chromosome 1p. We have demonstrated a preferred foetal expression of one transcript of the gene, which is in sharp contrast to its very weak or absent expression observed in neuroblastoma tumours. We have been able to show that *APITD1* in fact has cell growth and/or cell death properties in neuroblastoma cells by functional studies of the *APITD1*A mRNA in cell lines. Future studies of *APITD1* will be necessary to determine if it functions as a tumour suppressor gene upstream of *TP53* and whether mutations in *APITD1*, although not commonly present in neuroblastoma, might be of importance in other tumour types.
